# Iron, Biofilm, and Colorectal Cancer: Mechanistic Insights Into *Enterococcus faecalis* Pathogenesis

**DOI:** 10.1111/apm.70268

**Published:** 2026-10-02

**Authors:** Sibusiso Luthuli, Nathalie Scarpulla, Lorenzo Bertolino, Lulama Luthuli, Emanuele Durante‐Mangoni

**Affiliations:** ^1^ Department of Precision Medicine University of Campania ‘L. Vanvitelli’ Naples Italy; ^2^ Unit of Internal Medicine and Transplants Monaldi Hospital Naples Italy; ^3^ Department of Paediatric Surgery University of Pretoria, Kalafong and Steve Biko Academic Hospital of the University of Pretoria Pretoria South Africa

**Keywords:** biofilm formation, colorectal cancer, *Enterococcus faecalis*, extracellular DNA, iron homeostasis

## Abstract

*Enterococcus faecalis* is a commensal of the human gastrointestinal microbiota that has emerged as a successful opportunistic pathogen associated with persistent infections, antimicrobial resistance, and biofilm formation. Increasing evidence indicates that iron availability functions as a master environmental signal coordinating *E. faecalis* pathogenic traits. This review summarizes clinical, molecular, and sequencing‐based studies linking iron homeostasis to extracellular DNA (eDNA) dynamics, biofilm architecture, virulence regulation, and immune evasion, and critically evaluates the emerging but still associative evidence connecting *E. faecalis* to colorectal carcinogenesis. We highlight how iron sensing and uptake systems modulate biofilm stability through eDNA release, metabolic cooperation, and transcriptional control via the ferric uptake regulator (Fur) and other regulatory networks. We further integrate experimental findings that suggest how these mechanisms may promote inflammation‐driven tumorigenesis in the colon, although a complete causal pathway has not yet been established. Finally, we discuss translational implications, including iron‐targeting and eDNA‐disruptive strategies as promising anti‐virulence approaches, and identify key knowledge gaps requiring future investigation. This review positions iron metabolism at the center of *E. faecalis* pathogenicity and underscores its potential as a therapeutic target, while recognizing the need for further mechanistic and clinical validation.

## 1 Introduction

Enterococci are common commensals of the gastrointestinal tract that have emerged as significant nosocomial pathogens since the 1970s, ranking among the top three causes of healthcare‐associated infections [1]. *E. faecalis* is particularly formidable due to its ability to form robust biofilms, structured communities encased in an extracellular matrix that confer enhanced antimicrobial resistance and immune evasion [2].

The biofilm matrix comprises polysaccharides, proteins, lipids, and extracellular DNA (eDNA), which serves both as a structural scaffold and a signaling molecule [3]. A pivotal environmental cue coordinating *E. faecalis* pathogenesis is iron availability. In vivo, free iron is severely limited due to host sequestration, a condition termed nutritional immunity [4]. *E. faecalis* has evolved sophisticated mechanisms to sense and adapt to iron restriction, and this response is intrinsically linked to eDNA release and biofilm development [5, 6].

This review provides a mechanistic overview of the iron‐eDNA‐biofilm axis in *E. faecalis* pathogenesis and critically evaluates the epidemiological and experimental evidence linking *E. faecalis* to colorectal cancer (CRC). We identify knowledge gaps, propose specific hypotheses for future investigation, and discuss translational implications. Although individual aspects of *E. faecalis* pathogenesis (biofilm formation, iron homeostasis, and CRC association) have been reviewed separately, this review attempts a novel approach by integrating these three themes.

## 2 Methodology

This review was conducted as a narrative synthesis. A literature search was performed using PubMed, Scopus, and Web of Science from inception to April 2026. Search terms included: “ *Enterococcus faecalis* ,” “iron homeostasis,” “biofilm,” “extracellular DNA,” “Fur regulator,” “colorectal cancer,” and “mixed‐species biofilm.” Given the heterogeneity of included studies, a formal meta‐analysis was inapplicable. Findings were therefore analyzed thematically.

## 3 Biofilm Formation in *Enterococcus faecalis*

*E. faecalis* biofilms exhibit a complex three‐dimensional architecture of exopolysaccharides, proteins, lipids, and eDNA, which together contribute to biofilm structure and can increase tolerance to antimicrobial agents and host immune responses [2]. Biofilm formation encompasses initial adhesion, maturation, and dispersion, with key proteins including Esp and Ebp pili contributing to biofilm formation and organization [7, 8, 9] (Figure 1). eDNA is a critical structural component, regulated by autolysis and extracellular proteases [2]. *E. faecalis* biofilms are clinically associated with nosocomial infections, including surgical‐site infections, catheter‐associated infections, and urinary tract infections, and can act as reservoirs for antimicrobial resistance [8, 9].

**FIGURE 1:**
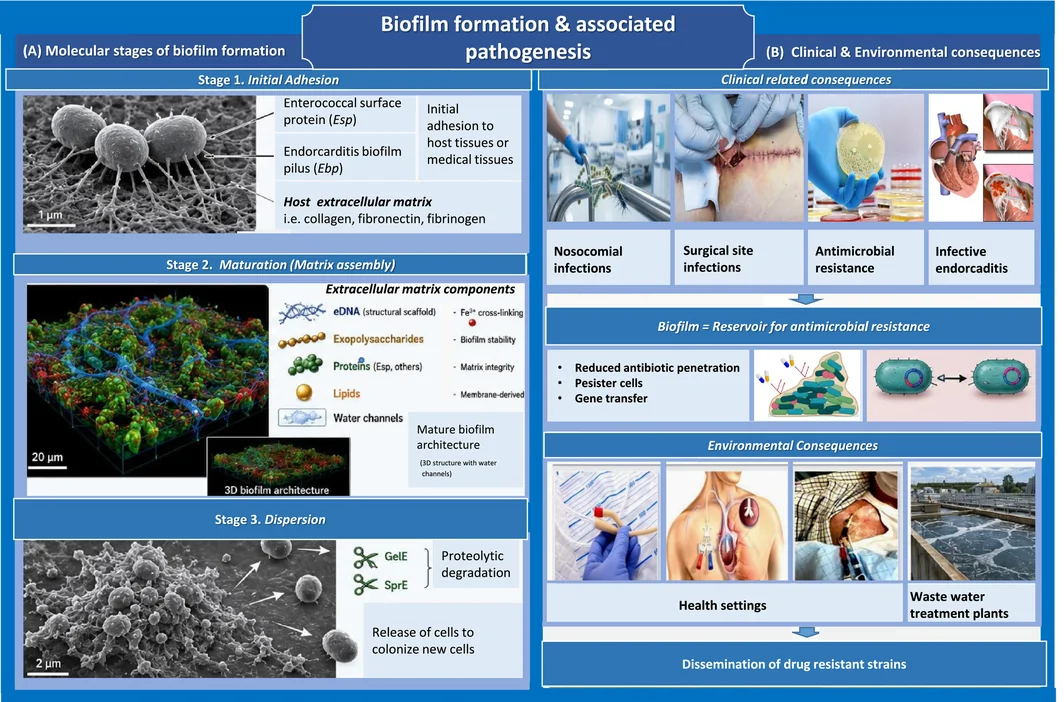
Biofilm formation and associated pathogenesis. (A) Molecular stages of biofilm formation. The process begins with initial adhesion mediated by the enterococcal surface protein Esp and Ebp pili, which facilitate binding to extracellular matrix proteins (collagen, fibrinogen, fibronectin) on host surfaces or medical devices. During maturation, the extracellular matrix assembles from eDNA, exopolysaccharides, proteins, and lipids, forming a three‐dimensional protective architecture. eDNA serves as a critical structural scaffold, cross‐linked by Fe^3+^ ions that enhance matrix rigidity and DNase resistance. Finally, dispersion is driven by proteolytic degradation (GelE, SprE), releasing cells to colonize new sites. (B) Clinical and environmental consequences. Biofilm formation is associated with nosocomial infections (urinary tract infections, bloodstream infections, infective endocarditis) and surgical site infections, particularly following colorectal resection. Biofilms act as reservoirs for antimicrobial resistance through reduced antibiotic penetration, persister cell formation, and horizontal gene transfer. Environmental contamination of water systems and healthcare settings facilitates the dissemination of drug‐resistant strains.

Biofilm development is influenced by nutrient availability and the capacity of bacterial cells to adapt to changing environmental conditions. Iron is one such element that may influence biofilm physiology, structure, and persistence.

## 4 Iron as a Modulator of Biofilm Formation

Iron serves as a cofactor for enzymes in metabolism, electron transport, and DNA synthesis, and *E. faecalis* demonstrates metabolic adaptability to varying iron levels, with changes in iron availability associated with alterations in biofilm characteristics [5]. Thus, beyond its role as an essential micronutrient, iron availability may influence the physiological and structural properties of *E. faecalis* biofilms.

### 4.1 Iron Availability and Biofilm Structure

Experimental variation in iron availability has been reported to alter biofilm biomass and structural characteristics in *E. faecalis* [5]. Iron has also been implicated in extracellular electron transfer (EET) within *E. faecalis* biofilms [10], which may contribute to metabolic activity across oxygen gradients and support cellular persistence in oxygen‐limited regions of the biofilm. Iron associated with the biofilm matrix has also been linked to EET‐dependent energy production and enhanced biofilm growth [10]. However, the extent to which EET directly determines overall biofilm stability or persistence remains to be further elucidated.

### 4.2 Iron Acquisition, Biofilm Persistence, and Virulence

The ability of *E. faecalis* to acquire iron under nutrient‐limited conditions is relevant to both growth and host‐associated persistence. The organism possesses five characterized iron uptake systems, EfaCBA, FeoAB, FhuDCBG, FitABCD, and EmtABC, which collectively contribute to iron acquisition and homeostasis [11]. Inactivation of all five systems in the Δ5Fe strain results in reduced iron uptake and impaired growth under iron‐depleted conditions, demonstrating functional overlap among the individual transport systems [11]. The contribution of these systems to virulence varies according to the infection model, indicating that their importance may be influenced by the host environment [11].

Heme provides an additional iron source when the characterized transport systems are disrupted [11]. Anaerobilin synthase (RS05575), an oxygen‐sensitive heme‐degrading enzyme, contributes to *E. faecalis* survival, gastrointestinal colonization, and virulence in experimental models [12]. Thus, the capacity to acquire and utilize heme in addition to other iron forms may support *E. faecalis* persistence in heterogeneous host environments.

### 4.3 The Colonic Iron Landscape

The colonic iron environment differs from standard in vitro conditions. Luminal iron derives from dietary residue, mucosal secretions, and desquamated epithelial cells, and its availability is shaped by pH, bacterial activity, and poorly soluble iron complexes. This heterogeneity may influence bacterial physiology and polymicrobial interactions.

### 4.4 Iron in Mixed‐Species Biofilms

*E. faecalis* and *Pseudomonas aeruginosa* ( *P. aeruginosa* ) frequently co‐occur in biofilm‐associated infections, yet their interactions under iron limitation remain relatively underexplored. Recent studies show that iron restriction enables *E. faecalis* to inhibit *P. aeruginosa* growth through increased L‐lactate production by the lactate dehydrogenase gene (*ldh1*). *Ldh1* expression is induced under iron limitation, promoting pyruvate‐to‐L‐lactate conversion; lactate export and dissociation acidify the local environment, while L‐lactate also chelates iron. The resulting decrease in pH and iron availability suppresses *P. aeruginosa* growth [13].

Conversely, in *E. faecalis* – *E. coli* biofilms, iron availability promotes the growth and persistence of both species, supporting *E. faecalis* despite interspecies competition [14]. These findings indicate that iron availability can influence interspecies interactions within biofilms and may contribute to differences in microbial growth and persistence under iron‐restricted or iron‐replete conditions.

The potential implications of manipulating iron availability or the local biofilm microenvironment are considered later in the context of translation.

## 5 Iron Homeostasis and Regulatory Networks in *Enterococcus faecalis*

### 5.1 The Fur Regulon and Iron‐Responsive Regulation

Latorre and colleagues characterized the *Fur* regulon in *E. faecalis* and showed that Fur regulates iron uptake genes including *feoAB*, *fhuDCBG*, and *yclQPN* [15]. Iron availability also altered broader transcriptional networks, including genes involved in transport, energy production, and Fe‐S proteins. Fur deletion increased intracellular iron and altered biofilm formation, indicating that iron homeostasis is linked to broader cellular adaptation [15].

These findings indicate that Fur contributes to iron homeostasis within a broader regulatory network through which *E. faecalis* responds to changes in iron availability, rather than functioning as an isolated regulator.

### 5.2 The Feo System: Ferrous Iron Uptake

The Feo system, comprising FeoA and FeoB, is one of the characterized mechanisms for ferrous iron (Fe^2+^) uptake in *E. faecalis* [11]. FeoB functions as the membrane‐associated component of the system, while FeoA serves as an accessory protein [11]. The *feoAB* operon is among the iron‐uptake systems associated with Fur‐dependent regulation [15]. Ferrous iron availability can be influenced by environmental factors such as oxygen tension and pH, which affect iron speciation and the forms of iron accessible to bacterial cells [14]. These characteristics may be relevant to iron acquisition under different host‐associated conditions, although the contribution of FeoAB to iron acquisition is best considered within the context of the multiple iron uptake systems present in *E. faecalis* .

### 5.3 Iron Uptake Machinery and Heme Utilization

Multiple iron uptake systems allow *E. faecalis* to respond to iron limitation through partially overlapping mechanisms [11]. The Δ5Fe phenotype demonstrates their collective importance for iron uptake and growth, while heme provides an additional iron source. RS05575‐mediated anaerobic heme degradation contributes to colonization and virulence in experimental models [11, 12].

### 5.4 Small Regulatory RNAs and Post‐Transcriptional Regulation

Beyond transcriptional control by Fur and other regulatory proteins, iron homeostasis in *E. faecalis* may also be influenced at the post‐transcriptional level by small non‐coding RNAs (sRNAs). Earlier work by Shioya et al. [16] identified several sRNAs in *E. faecalis* , including EFA0080_EFA0081 and EF0408_EF0409, providing evidence for an additional layer of RNA‐mediated regulation in this organism. Gómez‐Contreras [17] identified four sRNAs whose expression responds to iron availability: ncRNA_2860, ncRNA_3091, ncRNA_6060, and ncRNA_3122. The first three were predicted to target the FeoB iron transporter, whereas ncRNA_3122 was predicted to target Hemolysin III [17].

Because these interactions are still under investigation, their precise regulatory effects remain to be fully elucidated. Nevertheless, the iron‐responsive expression of these sRNAs and their predicted association with iron acquisition and related cellular functions suggest that sRNAs may contribute to iron homeostasis in *E. faecalis* . Together with transcriptional regulators and iron‐acquisition systems, sRNAs may therefore represent an additional regulatory layer through which *E. faecalis* responds to changing iron availability.

## 6 Mechanistic Evidence Linking Iron to Biofilm Formation & Persistence

The release of eDNA is a critical event in the initial adhesion and maturation of biofilms. Its role extends beyond structural support to include contributions to antimicrobial resistance and genetic exchange [6]. The regulatory pathways controlling eDNA release are complex, and emerging evidence indicates that environmental factors such as iron availability can influence this process.

A recent study by Zhen and colleagues examined this relationship under nutrient‐starvation conditions, providing mechanistic evidence that iron availability can influence biofilm development through modulation of eDNA release [6]. In starved *E. faecalis* cultures, iron supplementation promoted biofilm formation and was accompanied by an increase in extracellular eDNA. Conversely, iron restriction using gallium(III) nitrate, an iron‐mimetic compound, reduced overall biofilm formation while increasing the proportion of eDNA within the remaining biofilm [6]. Further evidence for the structural importance of eDNA came from DNase I treatment, which reduced eDNA content and viable bacterial cells in iron‐supplemented biofilms [6]. These observations support a model in which iron availability influences biofilm development partly through changes in eDNA release and matrix organization [6] (Figure 2). However, the study was performed under a defined starvation model, and therefore the extent to which the same mechanism operates across different host‐associated environments remains to be well established. Nevertheless, the findings provide a mechanistic link between iron availability, eDNA dynamics, and biofilm development that complements earlier observations.

**FIGURE 2:**
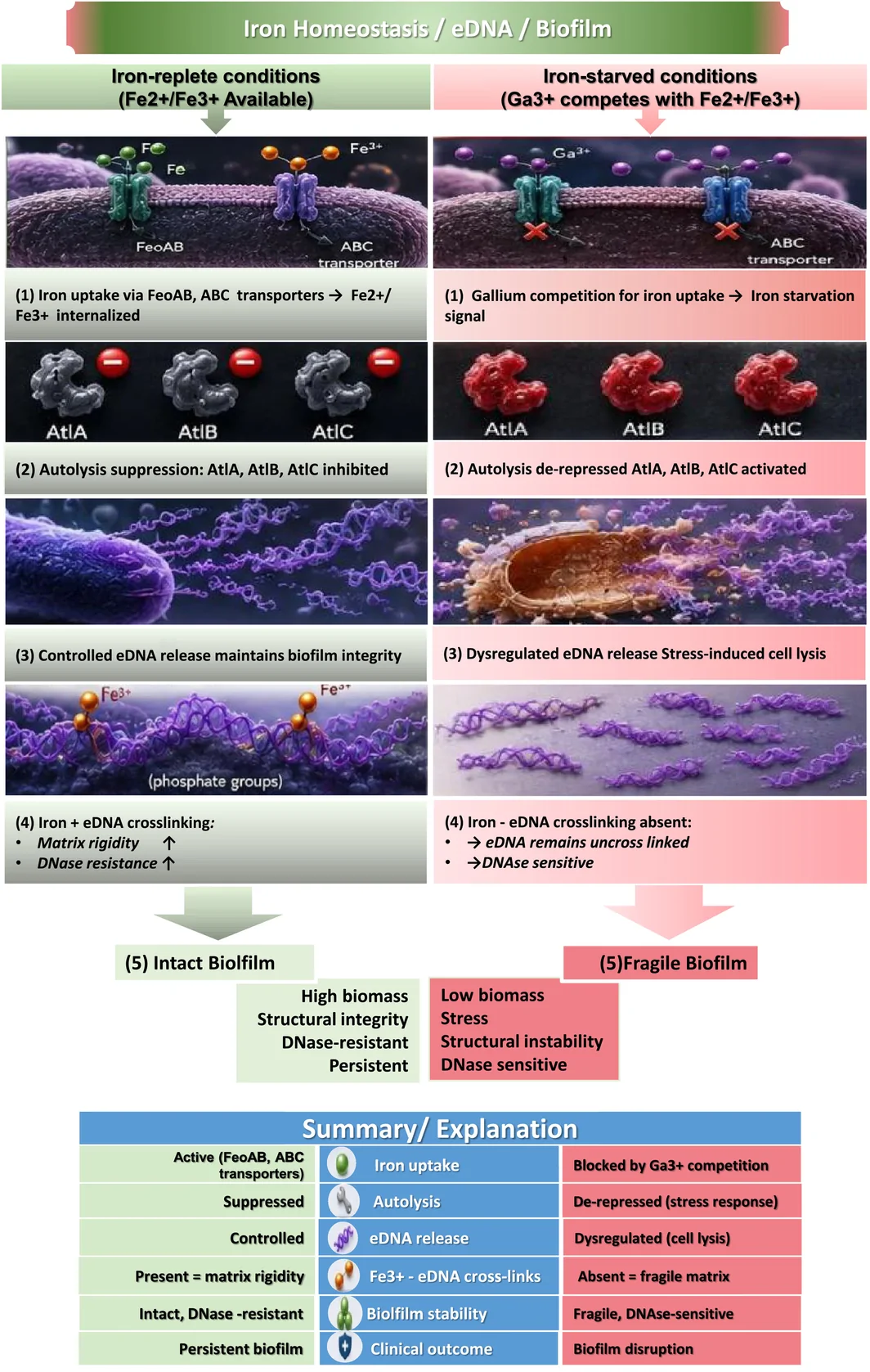
Iron homeostasis regulates eDNA release and biofilm integrity. Left panel (Iron‐Replete Conditions): In the presence of iron (Fe^2+^/Fe^3+^), *E. faecalis* actively imports iron via the FeoAB system and ABC transporters. Autolysis is suppressed through regulation of autolytic enzymes (AtlA, AtlB, AtlC), resulting in controlled eDNA release that supports biofilm integrity. Extracellular Fe^3+^ binds to phosphate groups on eDNA, forming Fe^3^‐eDNA cross‐links that increase matrix rigidity and confer resistance to DNase I degradation. The resulting biofilm is robust, structurally intact, and persistent. Right panel (Iron‐Starved Conditions): Gallium (Ga^3+^) competes with iron for uptake systems, inducing an iron starvation signal. This triggers a stress response that derepresses autolytic enzymes (AtlA, AtlB, AtlC), leading to dysregulated cell lysis and uncontrolled eDNA release. In the absence of Fe^3+^, eDNA cross‐linking does not occur, resulting in a structurally fragile, DNase‐sensitive biofilm. This demonstrates iron's dual role: As a nutrient for biomass accumulation and as a critical signal for eDNA scaffold stabilization.

Although not focused specifically on *E. faecalis* , the study by Sánchez‐Jiménez et al. [18] examined iron‐targeting approaches in *P. aeruginosa* . Their investigation considered multiple strategies for disrupting bacterial iron acquisition and utilization, including iron chelators such as lactoferrin, iron mimics such as gallium, siderophore‐antibiotic conjugates such as cefiderocol, and vaccines targeting iron transporters, several of which remain under active investigation. These approaches further highlight the therapeutic potential of the paradigm identified by Zhen and colleagues [6] by demonstrating that manipulation of iron availability or iron acquisition can be exploited as an anti‐infective strategy in another clinically important biofilm‐forming pathogen. Importantly, while the findings from *P. aeruginosa* cannot be directly extrapolated to *E. faecalis* , their mechanistic convergence with the observations described above provides a rationale for investigating whether iron‐targeting strategies could similarly disrupt *E. faecalis* biofilm establishment, maturation, or persistence. Such findings, viewed alongside the extensive body of research on *P. aeruginosa* , therefore support further investigation of iron‐targeting strategies as a potential component of future anti‐infective regimens, particularly for biofilm‐associated and recurrent infections in which conventional antibiotics may be less effective [18].

Govindarajan and colleagues found that Fe^2+^ concentrations of 50–150 μM promoted *E. coli* colonization, while 50–200 μM enhanced *E. faecalis* growth and dual‐species colony formation. Iron supplementation increased intracellular Fe^2+^ approximately fourfold in single‐species and elevenfold in dual‐species models and was also associated with increased *E. faecalis* antibiotic resistance [14]. The study also investigated the possible involvement of the FeoB ferrous iron transporter using homology modeling and molecular docking. The modeling suggested an interaction between FeoB and extracellular Fe^2+^, consistent with a possible role for FeoB in ferrous iron uptake. However, because these observations were derived from in silico modeling rather than direct experimental measurement of FeoB‐mediated transport, they should be interpreted as supportive rather than definitive evidence of the molecular mechanism [14].

Taken together, these findings highlight the importance of considering iron availability within the ecological context in which *E. faecalis* exists. In single‐species models, iron availability can influence biofilm formation and eDNA‐associated matrix organization, while in dual‐species systems, iron can additionally affect bacterial growth, intracellular iron accumulation, and antibiotic resistance. The effects of iron are therefore likely to depend not only on its concentration but also on nutritional status, community composition, and the physiological state of the participating organisms.

## 7 Iron, Heme, and the Colorectal Micro‐Environment

The relevance of iron to CRC extends beyond microbial nutrition. Dietary heme can alter the intestinal environment through lipid peroxidation, reactive aldehydes, endogenous N‐nitroso compounds, and DNA‐alkylating damage, increasing epithelial cytotoxicity and mutagenic stress [19]. Iron may also enhance WNT signaling in APC‐mutant cells [19]. Heme‐associated microbial changes and barrier disruption may increase exposure to microbial products and contribute to inflammation and compensatory proliferation [19]. These observations do not show that dietary iron or heme directly drives *E. faecalis* pathogenicity, but they identify a host environment in which microbial iron acquisition, epithelial iron handling, oxidative stress, and inflammation may intersect.

Heme exposure may also influence epithelial proliferation and selection of injury‐resistant cell populations. Iron can enhance WNT signaling in APC‐mutant cells [19]. Heme‐associated microbial alterations and barrier disruption may increase exposure to microbial products and luminal irritants, contributing to chronic inflammation and compensatory proliferation [19].

The intestinal microbiota and mucosal barrier provide another level of interaction. Heme‐associated microbial alterations and barrier disruption can increase exposure of epithelial cells to microbial products and luminal irritants, contributing to chronic inflammation and compensatory proliferation [19].

These observations do not demonstrate that dietary iron or heme directly drives *E. faecalis* pathogenicity. Rather, they establish a host environment in which microbial iron acquisition, epithelial iron handling, oxidative stress, and inflammatory responses may potentially intersect.

## 8 *Enterococcus faecalis* and Colorectal Carcinogenesis

Experimental evidence identifies several mechanisms by which *E. faecalis* may contribute to colorectal carcinogenesis, although causality varies. *E. faecalis* can produce extracellular superoxide and hydrogen peroxide, causing DNA damage in colonic epithelial cells [20]. Studies report DNA damage, γH2AX formation, aneuploidy, and tetraploidy, while in vivo hydroxyl radical production and extracellular superoxide‐associated chromosomal instability provide additional evidence of oxidative stress [20, 21, 22, 23].

Inflammatory responses provide another potential mechanism. *E. faecalis* can activate COX‐2, NF‐κB, and inflammatory cytokine pathways, and experimental models have linked colonization with colitis and dysplasia, particularly in genetically susceptible hosts [24]. Chronic inflammation may amplify epithelial proliferation and oxidative stress.

*E. faecalis* can affect epithelial barrier integrity through metalloproteases and gelatinase [25, 26]. Gelatinase‐mediated permeability and extracellular‐matrix remodeling may alter epithelial signaling, while strain‐specific effects on tumor formation and cancer‐cell migration have been reported [25, 27].

Additional mechanistic studies have shown that *E. faecalis* induces H_2_O_2_‐mediated epithelial cell death and enhances *Candida albicans* virulence. Gelatinase produced by *E. faecalis* mediates intestinal permeability via protease‐activated receptor 2 [25]. The bacterium also promotes a migratory and invasive phenotype in colon cancer cells [27]. Differential responses of colorectal cancer cell lines to *E. faecalis* strains isolated from healthy donors and CRC patients have been reported, indicating strain‐specific effects [28].

The role of *E. faecalis* in CRC is subject to debate. Some isolates have neutral or protective effects in experimental systems, and species‐level abundance may therefore be insufficient to predict biological behavior [28]. Increased abundance in CRC may reflect both the expansion of particular strains in the altered tumor environment and possible contributions of selected strains to epithelial stress and inflammation.

The driver‐versus‐passenger question therefore remains unresolved. Perhaps at this stage, with available findings and literature, “context” could be used as a guide to address the debate, based on the environment in which this bacterium presents under certain conditions. Therefore, further investigations are also needed to distinguish strain‐specific pathogenic effects from ecological expansion secondary to CRC‐associated changes in inflammation, barrier function, oxygenation, and nutrient availability.

## 9 Clinical Interface: Endorcarditis and Colorectal Neoplasia

The pathogenic potential of *E. faecalis* in CRC may extend beyond local tumor colonization. For instance, *E. faecalis* is also a primary cause of infective endocarditis, particularly in the elderly population with an underlying valvular disease or prior antibiotic exposure. This raises a compelling clinical question: Could enterococcal bloodstream invasion be a result of a destabilized colonic mucosal barrier caused by an occult neoplasm? Several studies have investigated this hypothesis. Pericas and colleagues tested this hypothesis in 2017 [29], in a retrospective study of 154 patients with *E. faecalis* infective endocarditis. In the subgroup with an unclear source of infection, 61 patients underwent a colonoscopy; 31 (51%) patients had a colorectal neoplasia, of which 5 had CRC (prevalence of 8.2%). The prevalence of colorectal neoplasia and CRC was higher than expected in the general population, and for this reason the authors recommended colonoscopy in this subgroup. Escolà‐Vergé and colleagues [30], extended the question to all patients with *E. faecalis* infective endocarditis (EFIE), regardless of whether a source of infection had been identified. Of 103 patients, 78 (76%) underwent a colonoscopy; 47 (60%) had endoscopic findings consistent with a possible portal of entry, and 39 of these (83%) had a colorectal neoplastic disease, including 2 cancers. In this study, the association between colorectal neoplasia and *E. faecalis* infective endocarditis was not confined to bacteremia of unexplained origin. Ursi and colleagues approached the question differently, comparing 85 patients with enterococcal infective endocarditis (82 due to *E. faecalis* , 2 to *E. durans*, 1 to *E. faecium* ) against 81 patients with *Streptococcus gallolyticus* infective endocarditis, a pathogen already established as a marker for colorectal neoplasia, as a control [31]. Of the 42 enterococcal infective endocarditis patients who underwent colonoscopy, 22 (52%) had colorectal neoplasia, including 7 cancers (17%), a rate statistically indistinguishable from the *S. gallolyticus* group. In the CRC group, 5 out of 7 patients benefitted from surgical treatment of the neoplasia. A later letter from the same group argued that colonoscopy should not be deferred even in asymptomatic patients [32]. Across these three studies, the prevalence of colorectal neoplasia and cancer was consistently above what would be expected in the general population, making a reasonable case that altered colonic permeability underlies at least some *E. faecalis* bacteremia episodes. These retrospective studies carry inherent selection biases and limited generalizability from small cohorts, even in multicenter designs, underscoring the need for larger prospective studies with systematic screening to confirm this association [29, 30, 31, 32].

### 9.1 Surgical Context

Several case reports describe colorectal neoplasia in patients with *E. faecalis* infective endocarditis, predominantly involving left‐sided tumors (Table 1). Reported management varies with tumor stage, bleeding, cardiac stability, and patient factors. Khan et al. described a T1N0M0 sigmoid tumor treated by elective left hemicolectomy after infection control [33], while Ebrahimpour et al. reported a metastatic hepatic flexure tumor treated by palliative resection [34]. Additional cases have been reported by Alzayidati et al. [35], Amarnani and Rapose [36], and Rizkou et al. [37].

**TABLE 1:** Surgical spectrum in reported cases of EFIE‐associated CRC.

Study	Age/gender	Location (side)	Stage	Surgery	Timing	Intent
Khan et al. [33]	55/M	Sigmoid (L)	T1N0M0 (I)	Left hemicolectomy	Elective	Curative
Alzayidati et al. [35]	70/M	Sigmoid‐descending (L)	pT3N0M0 (IIA)	Left hemicolectomy	Emergency	Curative
Amarnani and Rapose [36]	86/M	Rectosigmoid (L)	No mets	LAR + colostomy	Delayed	Curative
Rizkou et al. [37]	69/M	Mid‐rectum (L)	cT4aN2b	Protective colostomy	Staged	Supportive
Rizkou et al. [37]	40/M	Upper rectum (L)	Not staged	None (deferred)	Deferred	Pending
Ebrahimpour et al. [34]	71/M	Hepatic flexure (R)	Stage IV (mets)	Partial colectomy	Not specified	Palliative

Abbreviations: L, left‐sided; LAR, low anterior resection; mets, metastases; *R*, right‐sided.

*E. faecalis* is also associated with surgical site infections after colorectal surgery, with reported incidence of 6%–46% [38]. Collagenolytic activity may contribute to anastomotic leakage [39], while adhesins, cytolysin, gelatinase, and biofilm formation support colonization and persistence [2, 7].

### 9.2 Biofilm Presence in Left Versus Right Colon

Comparative microbiome studies identified distinct microbial profiles in CRC and between proximal and distal tumors [40, 41]. Dejea and colleagues, found biofilms predominantly associated with proximal CRC and adenomas, with bacterial epithelial invasion [41]. Biofilm‐associated epithelial cells showed reduced E‐cadherin, increased permeability, and elevated IL‐6 and STAT3 activation. Similar findings in some healthy individuals raise the possibility that biofilms may precede tumor development. Subsequent work linked biofilms with polyamine metabolism associated with CRC [42]. These findings support a possible role for biofilms in mucosal dysfunction and metabolic reprogramming, but causality remains uncertain.

The source of *E. faecalis* bacteremia is often unidentified. In patients with unexplained bacteremia or endocarditis, colonoscopy may identify occult colorectal neoplasia, although the optimal clinical indications require prospective evaluation.

### 9.3 Insights From Next‐Generation Sequencing

The enrichment of *E. faecalis* in CRC, including early‐onset disease [43], is associative and requires mechanistic validation. Metagenomic studies are vulnerable to classification and contamination errors, so species confirmation, appropriate databases, and rigorous negative controls are important. Future studies should integrate strain‐resolved multi‐omics with functional and mechanistic models to distinguish bacterial drivers from passengers.

Prospective cohorts should also clarify whether *E. faecalis* bloodstream infection is independently associated with colorectal neoplasia and whether specific microbial or clinical features identify patients who may benefit from colorectal evaluation. The experimental models and systems are summarized in Table 2 and Figure 3.

**TABLE 2:** Investigations linking *Enterococcus faecalis* to Colorectal Cancer.

Evidence type	Key findings	Models/systems	Strengths	Limitations	References
Epidemiological	Increased *E. faecalis* abundance in CRC; 41% CRC/adenoma in EFIE	Human cohorts	Clinical relevance	Cannot establish causality	[29, 30, 31, 40]
In vitro	*E. faecalis* produces ROS/H_2_O_2_ causing DNA damage	Colonic epithelial cell lines	Mechanistic insights	Simplistic context	[20, 22, 23]
Animal models	*E. faecalis* colonization promotes colitis/dysplasia	Mouse models (AOM/DSS)	In vivo relevance	Host‐specific	[24]
Shotgun metagenomics	*E. faecalis* virulence genes enriched in CRC strains	Human stool/tissue	Strain‐level resolution	Cannot establish causation	[40]
Metatranscriptomics	Active *E. faecalis* virulence gene expression in tumors	Human tumor tissues	Confirms gene activity	Correlation ≠ causation	[43]
Host transcriptomics	*E. faecalis* associated with NF‐κB/STAT3 activation	Human colonic cells	Identifies host pathways	Correlation ≠ causation	[42]

**FIGURE 3:**
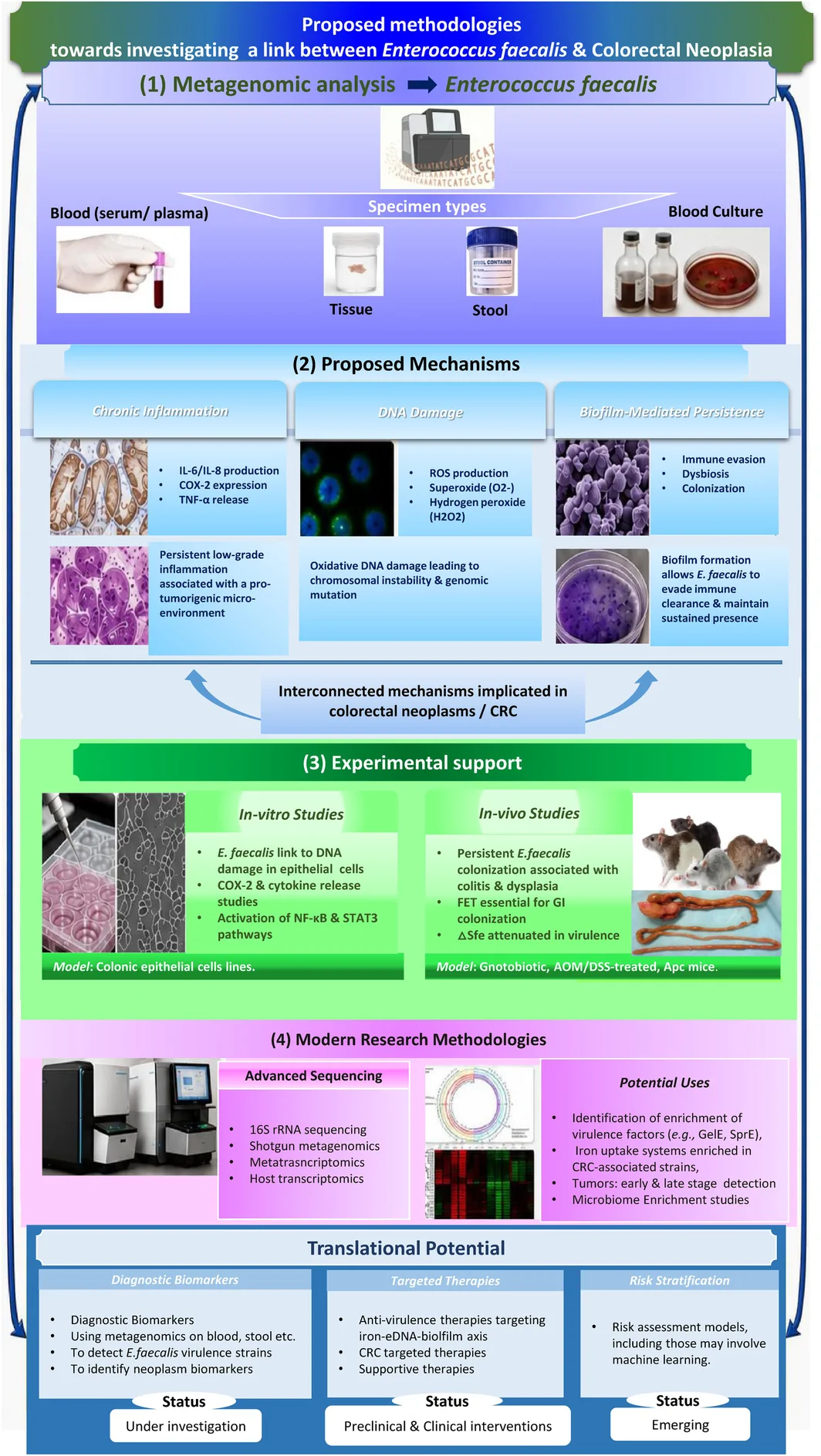
Proposed link between *E. faecalis* and CRC. Framework model integrating clinical, experimental, and genomic evidence illustrating the potential role of *E. faecalis* in colorectal carcinogenesis. Clinical observation: Metagenomic studies identify increased *E. faecalis* levels in blood, stool, and tissue samples of CRC patients. Proposed mechanisms: *E. faecalis* may contribute through three interconnected pathways: (1) chronic inflammation (IL‐6/IL‐8 production, COX‐2 expression, TNF‐α release) creating a pro‐tumorigenic microenvironment; (2) oxidative DNA damage via ROS production (superoxide and hydrogen peroxide) leading to chromosomal instability and genomic mutations; and (3) biofilm‐mediated persistence enabling immune evasion and sustained colonization. Experimental support: In vitro studies confirm *E. faecalis* causes DNA damage and inflammation in epithelial cells, while animal models demonstrate that persistent *E. faecalis* colonization exacerbates colitis and promotes tumorigenesis. Modern research context: Advanced sequencing techniques (16S rRNA, shotgun metagenomics, metatranscriptomics, host transcriptomics) are crucial for dissecting strain‐specific virulence and host responses. Translational Potential: This knowledge base enables the development of targeted anti‐virulence therapies (iron‐eDNA‐biofilm axis) and machine learning‐based risk stratification models for CRC.

## 10 Bridging the Iron‐eDNA‐Biofilm Axis With CRC Biology

The evidence reviewed above supports several interacting mechanisms but does not establish a single causal chain. *E. faecalis* can generate oxidative stress and induce epithelial DNA damage and chromosomal instability [20, 21, 22, 23], while iron can participate in redox chemistry reactions. It is therefore biologically plausible that local iron availability could influence oxidative stress at the microbial‐host interface.

However, there is no direct evidence that iron acquired through *E. faecalis* iron transport systems increases epithelial genotoxicity. The established observations that *E. faecalis* generates oxidative stress and that iron participates in redox chemistry should not be interpreted as evidence for a complete microbial iron‐dependent genotoxic pathway.

Biofilm formation provides another potential point of convergence. eDNA contributes to *E. faecalis* biofilm structure, while iron availability can influence biofilm biomass and matrix organization [6, 44, 45]. An iron‐responsive, eDNA‐rich biofilm could theoretically prolong bacterial persistence and microbial‐host contact at the mucosal interface.

Whether such biofilms alter local iron distribution, redox conditions, epithelial exposure, or inflammatory signaling in the colon remains unknown. Likewise, whether CRC‐associated environmental changes favor iron‐dependent biofilm phenotypes has not been established.

The appropriate interpretation is therefore a hypothesis‐generating model rather than an established mechanism linking intestinal iron availability, *E. faecalis* iron adaptation, biofilm/eDNA phenotypes, persistence, and epithelial stress. Each interface is supported to varying degrees, but the complete pathway remains uninvestigated.

## 11 Translational Implications

The iron‐eDNA‐biofilm framework suggests potential anti‐virulence strategies. Iron competition with gallium can inhibit biofilm development under starvation conditions, while DNase I can reduce eDNA‐associated biofilm structure [6]. These observations provide proof of concept for targeting microbial metal homeostasis or the biofilm matrix, but experimental findings cannot be directly extrapolated to clinical treatment.

Iron‐targeting strategies have also been investigated in *P. aeruginosa* using iron mimics, chelators, siderophore interference, and related approaches [18]. These studies provide a conceptual framework rather than direct evidence for therapeutic efficacy against *E. faecalis* .

The CRC implications remain preliminary. If microbial iron homeostasis, biofilm persistence, and epithelial stress are functionally interconnected, manipulating one component could theoretically influence another. However, there is insufficient evidence to recommend microbial iron manipulation or eDNA‐targeting approaches for CRC prevention or treatment [19].

Iron‐related pathways within the tumor represent a separate therapeutic field. Transferrin, transferrin saturation, transferrin receptor 1, ferritin, DMT1, and other iron‐regulatory pathways have been investigated as biomarkers or therapeutic targets in CRC [19]. Their associations with outcome are context‐dependent, and systemic iron status should not be equated with microbial or tumor‐local iron availability.

Tumor‐associated iron biology has also been explored through ferritin‐based nanocarriers and transferrin‐directed delivery systems. These approaches differ from microbial iron‐targeting strategies but illustrate the broader therapeutic relevance of iron metabolism.

## 12 Future Perspectives

Several questions require investigation. The relationship between Fur‐dependent iron sensing and eDNA release should be examined directly under physiologically relevant iron‐starvation conditions. Strain‐resolved studies are needed to determine whether CRC‐associated *E. faecalis* isolates possess distinctive iron acquisition, redox, biofilm, or proteolytic phenotypes.

The iron‐biofilm relationship should be studied in intestinal models that reproduce relevant oxygen gradients, nutrient limitation, host‐derived iron sources, and polymicrobial communities. Experiments should also distinguish effects of microbial iron acquisition from those of extracellular iron and host iron metabolism.

Prospective clinical studies are needed to determine whether EFIE is independently associated with colorectal neoplasia and which clinical or microbiological features identify patients most likely to benefit from colorectal evaluation. The EnteroColonus‐GAMES protocol provides a framework for larger clinical cohorts and mechanistic studies before translation into routine practice [46].

## 13 Conclusion

Iron represents an important intersection between *E. faecalis* physiology, biofilm persistence, and colorectal biology. Multiple iron acquisition systems and regulatory mechanisms support adaptation to iron limitation, while experimental evidence indicates that iron availability can influence biofilm formation, eDNA‐associated matrix organization, microbial competition, and antibiotic tolerance.

Selected *E. faecalis* strains can generate oxidative stress, epithelial DNA damage, chromosomal instability, inflammation, and tissue remodeling. Clinical studies have also identified colorectal neoplasia in subsets of patients with *E. faecalis* infective endocarditis, although the strength and specificity of this association remain uncertain.

The available evidence does not establish a linear iron‐ *E. faecalis* ‐biofilm‐CRC pathway. Instead, it identifies interacting interfaces involving microbial iron homeostasis, eDNA‐dependent biofilm persistence, epithelial oxidative stress, inflammation, mucosal integrity, and host iron metabolism. The priority is to investigate these processes in physiologically relevant, strain‐resolved models.

Such studies may determine whether iron‐dependent biofilm phenotypes are primarily persistence mechanisms or whether they modify *E. faecalis* ‐host interactions in colorectal disease. They may also clarify whether microbial iron homeostasis, eDNA dynamics, or specific virulence phenotypes could serve as biomarkers or anti‐virulence targets.

## Funding

The authors have nothing to report.

## Ethics Statement

The authors have nothing to report.

## Consent

The authors have nothing to report.

## Conflicts of Interest

The authors declare no conflicts of interest.

## Data Availability

The authors have nothing to report.
